# Tumor treating fields in malignant pleural mesothelioma: from cytoskeletal collapse to immunophenotypic conversion

**DOI:** 10.3389/fimmu.2026.1782708

**Published:** 2026-05-26

**Authors:** Xiaofeng Ma, Zhuo Zuo, Yulong Sun, Hong An

**Affiliations:** 1 Emergency Center, The Second Hospital of Lanzhou University, Lanzhou, Gansu, China; 2 School of Life Sciences and Technology, Key Laboratory for Space Biosciences & Biotechnology, Institute of Special Environmental Biophysics, Research Center of Special Environmental Biomechanics and Medical Engineering, Engineering Research Center of Chinese Ministry of Education for Biological Diagnosis, Treatment and Protection Technology and Equipment, Northwestern Polytechnical University, Xi’an, Shaanxi, China

**Keywords:** immunogenic cell death, malignant pleural mesothelioma, physio-immunological remodeling, tumor microenvironment, tumor treating fields

## Abstract

The therapeutic efficacy in malignant pleural mesothelioma (MPM) is severely hindered by its dense stromal barrier and immunosuppressive microenvironment. Although Tumor Treating Fields (TTFields) have demonstrated significant survival benefits in clinical trials, their underlying mechanisms extend well beyond simple mitotic arrest. This review delineates a “multidimensional biophysical remodeling” atlas of TTFields in MPM. First, we highlight that TTFields physically disrupt actin-dependent intercellular “tunneling nanotubes, ” thereby severing the tumor’s communication and metabolic rescue networks. Second, physical stress downregulates the Fanconi Anemia-BRCA pathway, inducing a state of “conditional BRCAness” that specifically sensitizes tumors to DNA-damaging agents. Finally, this cascade triggers immunogenic cell death and orchestrates a chemokine storm, which is hypothesized to facilitate the conversion of the tumor microenvironment from an immune “cold” to a “hot” phenotype. Collectively, by integrating the physical disintegration of subcellular structures with immunological remodeling, TTFields may offer a hypothetical theoretical framework for overcoming therapeutic resistance in MPM.

## 1 Introduction

### 1.1 Current treatment landscape and challenges

Malignant pleural mesothelioma (MPM) is a highly invasive tumor originating from pleural mesothelial cells, closely associated with asbestos exposure and characterized by an inferior prognosis. Over the past few decades, the exploration of systemic treatments for advanced unresectable MPM has been fraught with challenges (1). Since 2003, based on the pivotal phase III clinical trial results by Vogelzang et al., pemetrexed combined with cisplatin has established itself as the first-line standard chemotherapy regimen for MPM, improving the median overall survival (OS) to approximately 12.1 months (2). In the subsequent decade, although researchers attempted various improvement strategies—such as the MAPS study confirming that the addition of the anti-angiogenic agent bevacizumab to chemotherapy could further extend survival to 18.8 months—such benefits were often accompanied by increased systemic toxicities like hypertension and thromboembolism, failing to fundamentally alter the refractory nature of MPM (3). In recent years, with the introduction of immune checkpoint inhibitors (ICIs), the CheckMate 743 phase III clinical trial established that nivolumab combined with ipilimumab is a new standard of care for first-line treatment, significantly improving survival in patients with non-epithelioid subtypes (4). However, clinical practice has revealed that the complex tumor microenvironment (TME) and the high histological heterogeneity of MPM—manifested as a continuum from epithelioid to sarcomatoid subtypes (5)—lead to poor immunotherapy responses in some patients. In particular, the dense stromal barrier and the “immune-cold” characteristics, characterized by the absence of effector T-cell infiltration, remain key bottlenecks limiting further efficacy improvements.

### 1.2 The intervention of a physical modality: clinical breakthrough of tumor treating fields

At a time when pharmacological treatments are encountering bottlenecks, Tumor Treating Fields (TTFields), as a non-invasive, purely physical treatment modality, offer an entirely new dimension for the comprehensive treatment of MPM. TTFields act directly on the tumor region by delivering alternating electric fields at a specific frequency (optimized to 150 kHz for MPM) and low intensity (1–3 V/cm). Data from the single-arm phase II STELLAR clinical trial showed that TTFields, combined with standard first-line chemotherapy, resulted in a median OS of 18.2 months in patients with unresectable MPM, without significantly increasing chemotherapy-related systemic toxicity, which manifested only as manageable local skin reactions (6). Based on this encouraging clinical evidence, TTFields have been approved for the clinical treatment of MPM. Unlike biochemical drugs that rely on receptor binding or metabolic pathways, TTFields use physical field effects to interfere with polar molecules within cancer cells; this unique mechanism of action endows them with the potential to be combined with various therapies, such as chemotherapy and immunotherapy.

### 1.3 Deepening mechanistic understanding: multidimensional remodeling beyond mitosis

Although the clinical efficacy of TTFields has been preliminarily validated, the understanding of their biological mechanisms has long been confined to the “anti-mitotic” theory. Classical models posit that TTFields primarily disrupt spindle assembly by interfering with tubulin polymerization and generate dielectrophoretic forces at the cleavage furrow due to field inhomogeneity, leading to structural destruction and mitotic catastrophe (7, 8). However, mitotic arrest alone cannot fully explain the broad biological effects of TTFields observed clinically, particularly their synergy with DNA-damaging agents and immunotherapy. The latest preclinical studies have begun to reveal a more complex mechanistic landscape: TTFields can induce “conditional BRCAness” by downregulating the Fanconi Anemia-BRCA (FA-BRCA) pathway, thereby specifically increasing sensitivity to DNA-damaging agents such as cisplatin (9); furthermore, they can physically block actin-dependent “tunneling nanotubes (TNTs)”, severing the material rescue and signal communication networks between tumor cells (10).

### 1.4 Perspective of this review: constructing a multidimensional framework of parallel biological modules atlas

**Figure 1:**
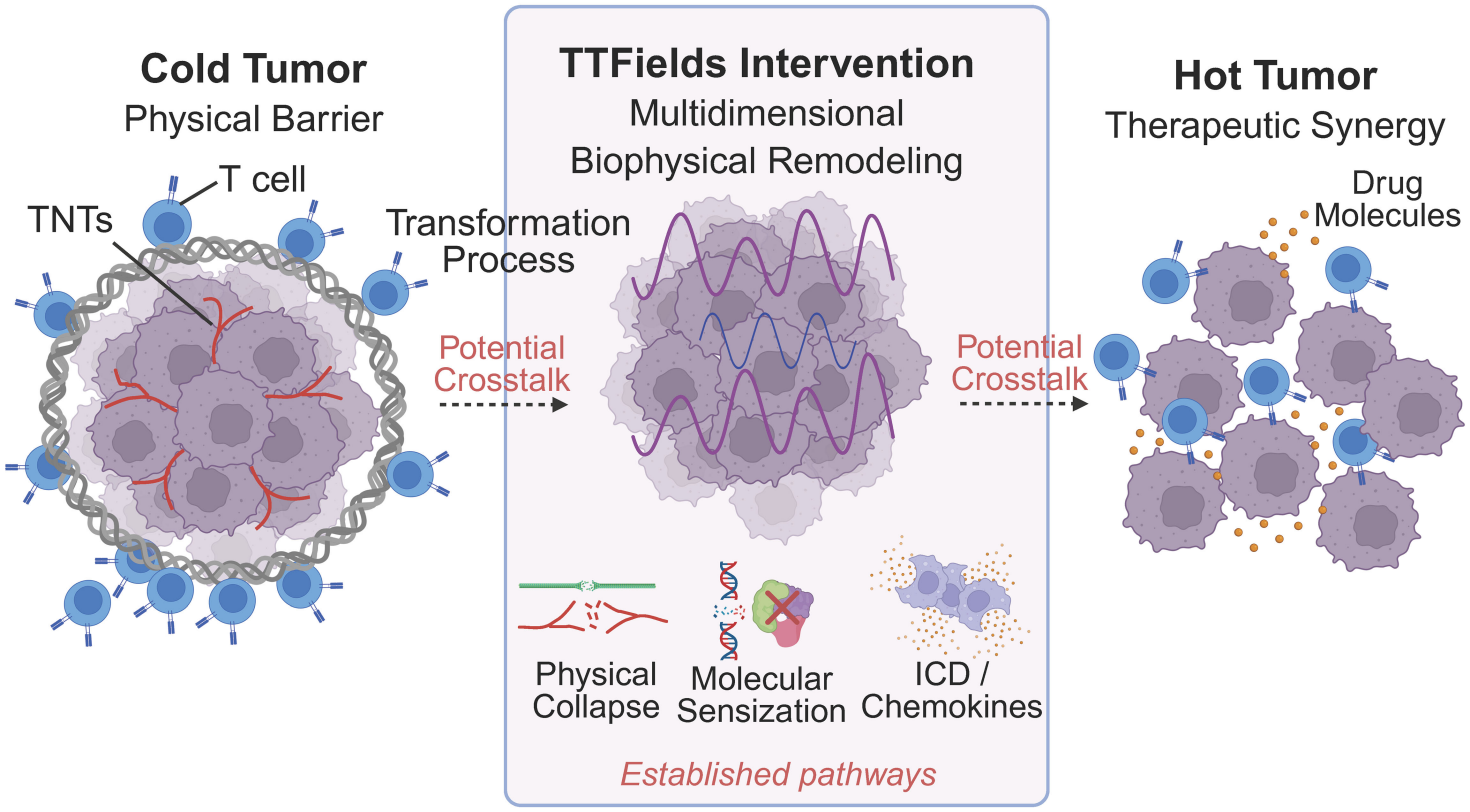
The physio-biological remodeling atlas driven by TTFields in MPM. This review constructs a multidimensional mechanistic atlas of TTFields in MPM. TTFields remodel the therapeutic landscape via a three-tier cascade: (1) Physical Strike: Disrupting mitotic spindles and severing intercellular tunneling nanotubes; (2) Molecular Sensitization: Downregulating the FA-BRCA pathway to induce “conditional BRCAness”, sensitizing tumors to DNA-damaging agents; (3) Ecological Remodeling: Inducing immunogenic cell death (ICD) and chemokine release, converting the “cold” TME into a “hot” phenotype. (Middle parts indicate mechanisms supported by current in vitro and in vivo preclinical evidence, while dashed arrows represent hypothetical inferences and emerging biological crosstalk that require further validation).

Based on the aforementioned background, this review aims to construct a multidimensional therapeutic atlas integrating physical mechanics and molecular biology to systematically elucidate the “multidimensional framework of parallel biological modules remodeling” mechanism of TTFields in MPM (**Figure 1**). We will discuss the cascade effects across three levels: concurrently, how TTFields physically block intercellular communication networks by disrupting cytoskeletal networks, particularly actin-dependent “TNTs”; in parallel, exploring how physical stress induces the downregulation of the FA-BRCA pathway to construct a “conditional BRCAness” state for enhancing chemotherapy; and additionally, analyzing how TTFields drive the transformation of the immunosuppressive microenvironment into an active state supporting anti-tumor immunity through immunogenic cell death (ICD) and chemokine regulation. The establishment of this physio-biological coupling perspective will provide a scientific basis for optimizing TTFields application strategies within a multidisciplinary, comprehensive treatment of MPM.

## 2 The physical strike — cytoskeletal and structural collapse

**Figure 2:**
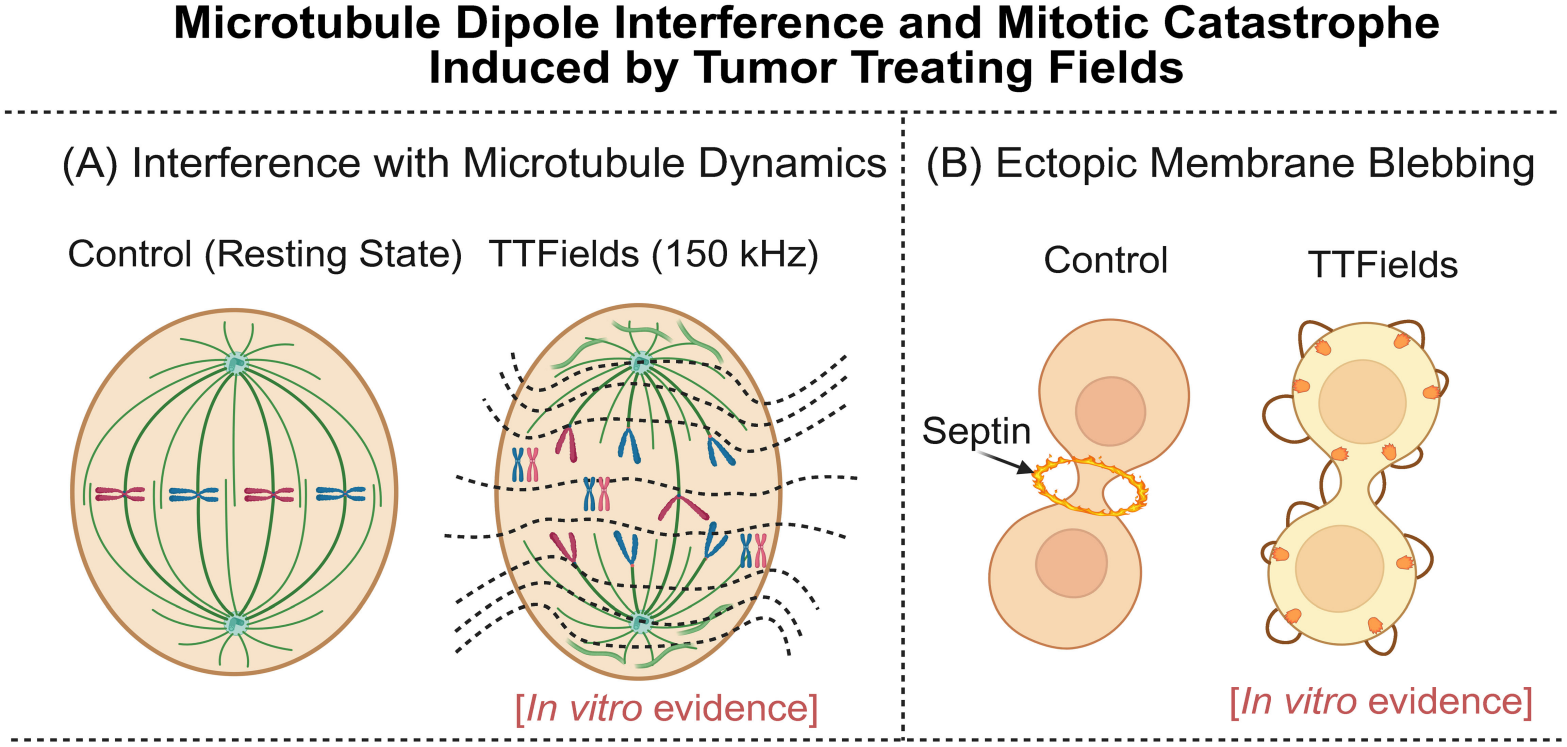
Microtubule dipole interference and mitotic catastrophe induced by tumor treating fields. **(A)** Interference with Microtubule Dynamics: In the resting state, tubulin dimers are randomly oriented. Upon application of 150 kHz alternating electric fields, tubulin dimers with high dipole moments are forced to align along the field lines, thereby inhibiting microtubule polymerization and disrupting spindle structure. **(B)** Ectopic Membrane Blebbing: During telophase, the highly non-uniform electric field at the cleavage furrow generates dielectrophoretic forces, causing mislocalization of Septin complexes (Septin 2, 6, 7). This prevents the formation of a regular ring structure at the cleavage furrow, triggering ectopic membrane blebbing and cytokinesis failure. (The described phenomena reflect observations derived primarily from in vitro experimental models).

The core therapeutic mechanism of TTFields is founded on the interaction between biophysical forces and subcellular structures. Unlike traditional pharmacological interventions that rely on ligand-receptor binding, TTFields utilize alternating electric fields to exert physical forces on macromolecules with high electric dipole moments within cancer cells, thereby disrupting the integrity of the cytoskeletal system. In MPM, this physical impact manifests as a cascading disintegration process, ranging from perturbing microtubule dynamics to disrupting intercellular communication networks (**Figure 2**).

### 2.1 Dipole interference of microtubules and mitotic catastrophe

Tubulin heterodimers are the fundamental units constituting the mitotic spindle and possess significant intrinsic electric dipole moments. Computer simulations and surface plasmon resonance studies indicate that alpha- and beta-tubulin monomers and dimers exhibit strong dielectric responses in electric fields, with dipole moments reaching |p_alpha_|=552D and |p_beta_|=1193D, respectively (11). Based on this physical property, when intermediate-frequency electric fields of 100–300 kHz are applied, tubulin dimers experience significant torque. This physical force aligns the dimers along the field lines. This direction often conflicts with the axial orientation required for typical spindle microtubule polymerization, thereby physically impeding microtubule assembly (8).

Numerical analysis models further reveal that the torque generated by TTFields during mitosis is sufficient to counteract the thermal energy of Brownian motion, thereby disrupting the spindle microtubule structure (12). In MPM cells and other solid tumor cells, this persistent physical interference leads to chromosome segregation abnormalities, multipolar spindle formation, and the inability to exit mitosis normally, ultimately triggering a state of “mitotic catastrophe” (7). Live-cell imaging technology has documented that under TTFields treatment, the duration of mitosis and cytokinesis is significantly prolonged, with some cells disintegrating directly during metaphase without entering the next cell cycle (13).

### 2.2 Mislocalization of the septin scaffold and cytokinesis failure

The physical disintegration of the cytoskeleton is not limited to the microtubule system. The Septin protein family (particularly Septin 2, 6, and 7) plays a critical scaffolding role at the cleavage furrow during cytokinesis, recruiting actin and myosin required for the contractile ring. Studies have found that TTFields can perturb the precise localization of Septin complexes at the cleavage furrow, causing them to form disordered aggregates on the cell membrane rather than organized ring structures (14). This structural misalignment directly leads to cytokinesis failure and ectopic membrane blebbing.

Biophysical model analyses indicate that during the late stages of cell division, the narrow cytoplasmic bridge at the cleavage furrow causes a sharp increase in current density, resulting in a highly non-uniform electric field distribution. The dielectrophoretic forces generated by this non-uniform field may further exacerbate the abnormal accumulation of polar macromolecules, such as Septin, at the cleavage furrow, thereby obstructing the physical process of cell separation (15).

### 2.3 Remodeling of the actin network and inhibition of cell motility

The disintegration of the microtubule system triggers a cascade of reactions in downstream signaling pathways, which in turn affect the conformation of the actin cytoskeleton. Evidence suggests that TTFields treatment leads to microtubule depolymerization, thereby releasing the microtubule-bound guanine nucleotide exchange factor GEF-H1. Released GEF-H1 activates the RhoA-ROCK signaling axis, leading to the abnormal formation of actin stress fibers and alterations in focal adhesions (16). This forced remodeling of the actin network significantly impairs cancer cell motility, as evidenced by reduced migration speed and inhibited podosome formation. This mechanism indicates that the physical strike effect of TTFields has extended beyond simple interference with cell division to inhibit cell motility.

### 2.4 Physical severing of intercellular communication: blocking tunneling nanotubes

**Figure 3:**
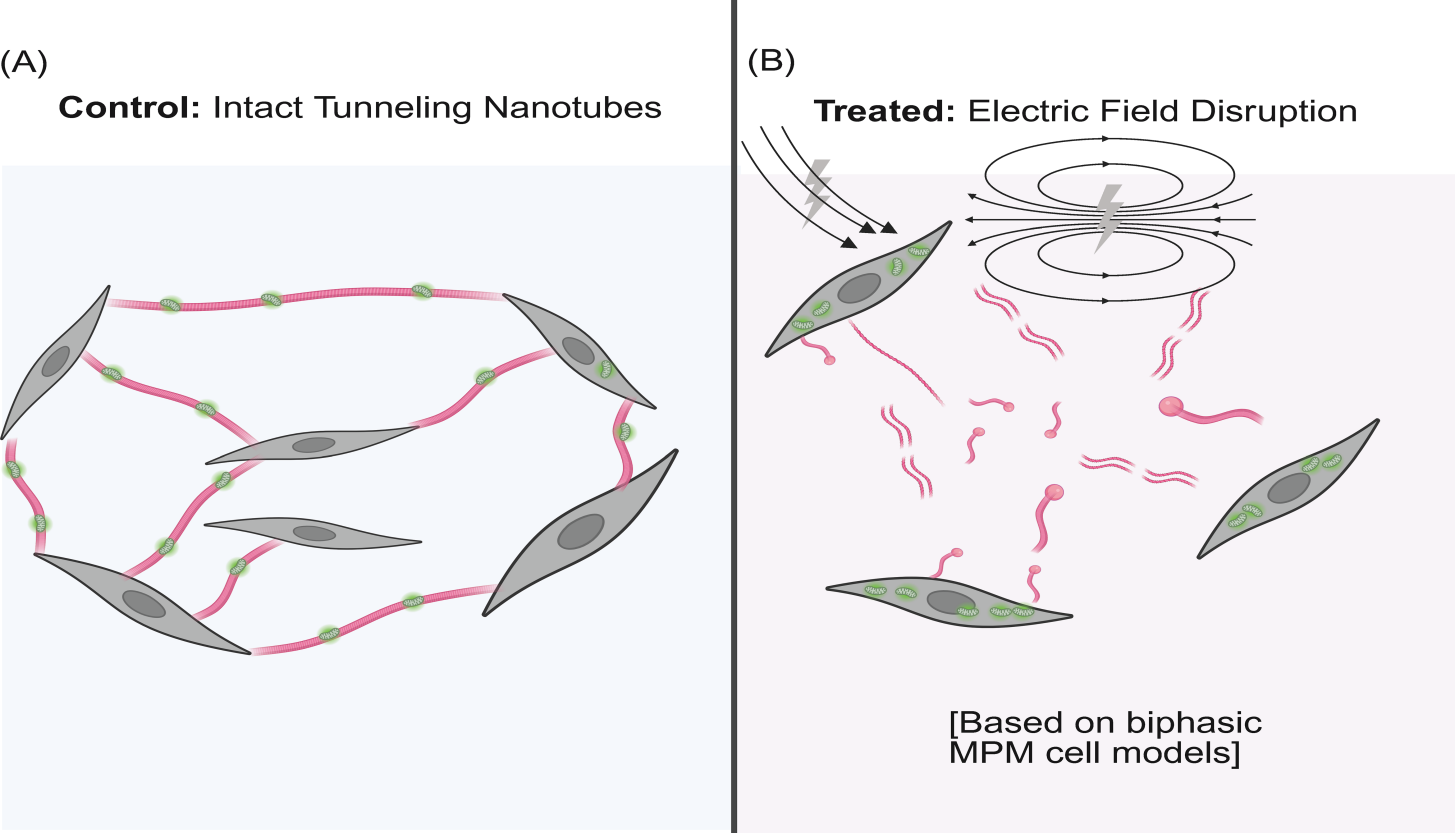
Physical severing of actin-dependent tunneling nanotubes by electric fields. This figure illustrates the extensive network of Tunneling Nanotubes between MPM cells. **(A)** Control: Biphasic MPM cells are connected by slender F-actin tubes, through which mitochondria (green) and KRAS oncogenic signals (red particles) shuttle freely for metabolic rescue. **(B)** Treated: Under 1.0 V/cm electric fields, remodeling of the actin cytoskeleton leads to rupture, retraction, or twisting of TNTs. Intercellular physical connections are severed, isolating damaged cells and preventing them from obtaining metabolic support from neighboring cells. (Current structural disruption evidence is based on in vitro biphasic MPM cell line studies).

In the TME of MPM, cancer cells do not exist in isolation but form complex intercellular communication networks via F-actin-based “TNTs”. These long-range membrane tubular structures have been increasingly observed and are suggested to be present in MPM, mediating the intercellular transfer of organelles such as mitochondria and Golgi apparatus, as well as cytosolic molecules (17). This physical connection allows damaged cells to acquire metabolic support from neighboring cells and even propagate mutant KRAS oncogenic signals, thereby promoting overall tumor survival and the spread of drug resistance (18) (**Figure 3**).

Recent research has revealed, for the first time, the destructive effect of TTFields on this communication structure. Experimental data show that the application of TTFields at 1.0 V/cm and 200 kHz significantly inhibits TNTs formation in biphasic MPM cells (e.g., MSTO-211H), resulting in a substantial decrease in intercellular connections (10). Although TTFields did not directly alter actin polymerization kinetics in cell-free systems, their inhibition of TNTs in living cells may be achieved by perturbing upstream cytoskeletal regulatory pathways. This physical blockade of TNTs severs the “rescue hotline” between tumor cells, rendering cancer cells under chemotherapy stress unable to obtain external metabolic buffering, thereby exacerbating treatment-induced cell death. Notably, the inhibitory effect of TTFields on TNTs exhibits particular cell subtype specificity, suggesting that intrinsic cytoskeletal stiffness or assembly dynamics may influence its physical effects (10).

The physical disruption of cytoskeletal networks and tunneling nanotubes underscores the necessity of optimal dosimetric coverage in practice. Clinically, this highlights the importance of utilizing personalized Treatment Planning Systems (TPS) to ensure that continuous and uniform electric fields effectively encompass the highly heterogeneous pleural tumor bed, thereby maximizing the structural disintegration of the tumor’s metabolic rescue pathways.

While these findings by Sarkari et al. offer a novel perspective on intercellular communication disruption, it should be noted that direct evidence linking TTFields to TNT blockade in MPM remains limited to a few specialized models. Further independent investigations across diverse histological subtypes are required to confirm the universality of this structural impact.

The physical vulnerability of TNTs to TTFields can be further elucidated through dielectrophoresis modeling. Based on the dielectric properties of F-actin, mathematical simulations indicate that intermediate-frequency electric fields exert a rotational torque on these high-aspect-ratio structures. Theoretical calculations (12) suggest that the dipole moment of actin monomers aligns with the field lines, which, in the case of TNTs spanning across non-uniform field gradients, generates a mechanical stress component. In a dense stromal environment where electric field lines are forced through narrow cytoplasmic or interstitial bridges, the resulting dielectrophoretic force may reach magnitudes capable of counteracting the intrinsic mechanical tension of the actin filaments, ultimately leading to structural fragmentation.

It is prudent to note that while the structural and functional aspects of TNTs are becoming increasingly characterized *in vitro*, their universality and overall clinical relevance in MPM remain subjects of active academic debate. The translation of these findings to a clinical setting is complicated by the dense, desmoplastic extracellular matrix inherent to pleural mesothelioma. This highly restrictive physical environment may naturally limit the extensive formation and maintenance of these fragile intercellular structures *in vivo*. Consequently, the degree to which TTFields-mediated TNT disruption translates into a substantial therapeutic benefit in patients presents a notable area of uncertainty. Future translational research is expected to address these spatial and biomechanical constraints to clarify the true clinical impact of targeting TNT networks.

## 3 The molecular vulnerability — induced BRCAness

**Figure 4:**
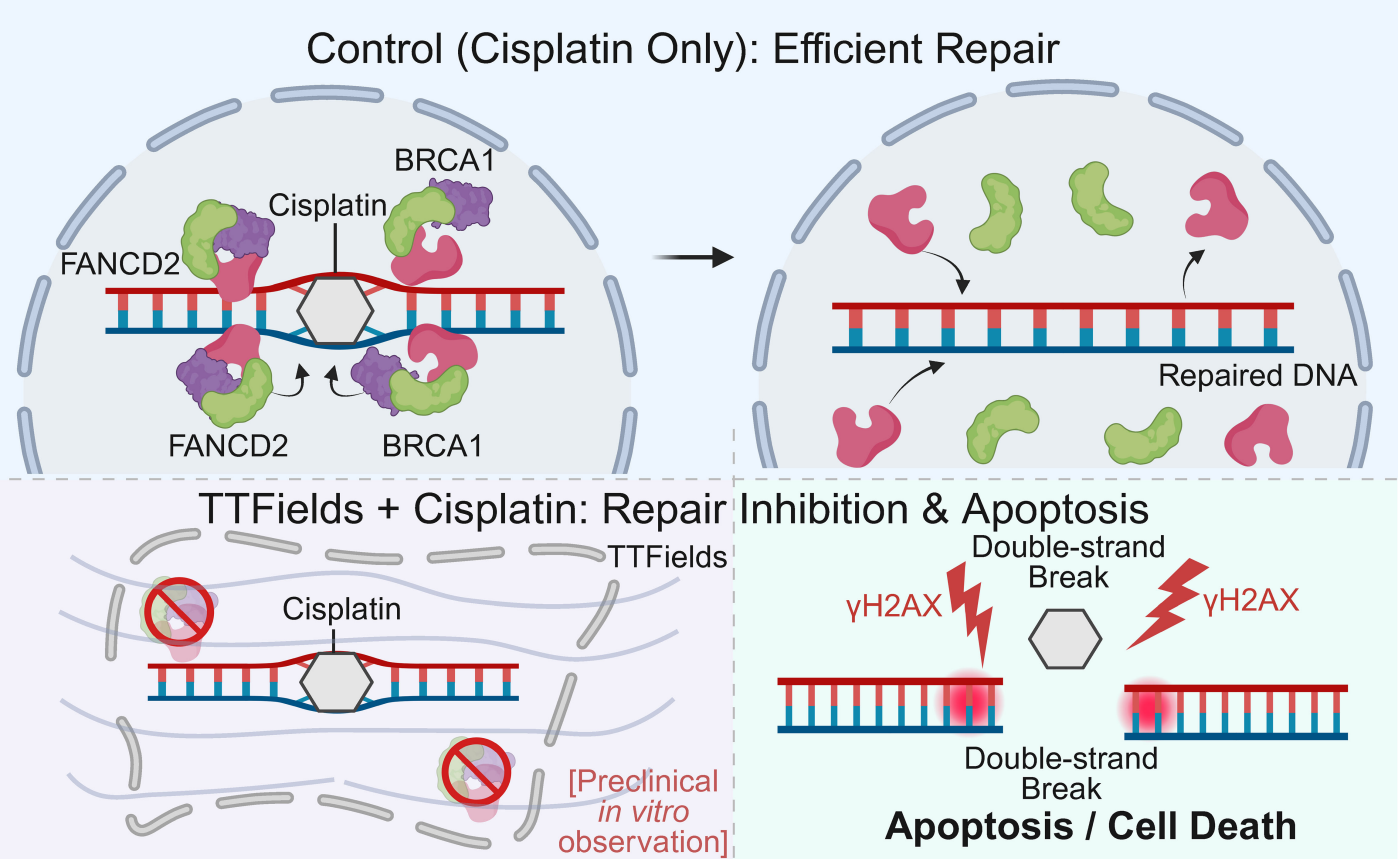
Induced BRCAness and synthetic lethality with platinum agents. **(A)** Normal Repair Pathway: Cisplatin-induced DNA interstrand cross-links (ICLs) typically activate the FA-BRCA pathway, with FANCD2 and BRCA1 proteins recruited to the damage site for repair. **(B)** Synthetic Lethality Pathway: TTFields-induced physical stress leads to significant downregulation of core FA-BRCA pathway proteins (FANCA, FANCD2, BRCA1). In the absence of repair proteins, cisplatin-induced damage remains unrepaired, leading to replication fork stalling and collapse, culminating in lethal DNA double-strand breaks (DSBs, marked by γH2AX), ultimately triggering apoptosis. (The downregulation of the FA-BRCA pathway illustrated here is currently supported by preclinical in vitro data).

The therapeutic efficacy of TTFields in MPM stems not only from structural disruption at the physical level but also involves a deeper mechanism concerning the systemic impairment of DNA damage repair (DDR) capabilities in cancer cells. Recent research evidence indicates that TTFields can specifically downregulate the FA-BRCA pathway, inducing a transient, non-genetic state of “BRCAness.” This remodeling at the molecular level provides a new biological basis for overcoming chemotherapy resistance in MPM, particularly by creating a “synthetic lethality” effect with DNA-damaging agents, significantly enhancing the potential of comprehensive treatment strategies (**Figure 4**).

### 3.1 Upstream triggers: from physical stress to replication stress

The molecular vulnerability induced by TTFields arises from their physical interference with cell division. As previously mentioned, the non-uniform electric fields and dielectrophoretic forces generated by TTFields at the cleavage furrow increase local energy density (15). This physical action not only directly interferes with microtubule dynamics but may also trigger intracellular ionic imbalance and metabolic stress by altering cell membrane potential and increasing membrane permeability (12).

Crucially, mitotic abnormalities caused by TTFields—including chromosome segregation errors and multinucleation—are direct drivers of intracellular replication stress (7). This persistent replication stress leads to the accumulation of DNA double-strand breaks, as evidenced by a significant increase in γH2AX foci within the nucleus (9). The nuclear fragmentation and apoptotic body formation observed in early studies are morphological manifestations of this DNA damage accumulation exceeding the cellular tolerance threshold (8). Thus, TTFields act as both a physical disruptor of cellular structure and a disturber of genomic stability.

### 3.2 Core mechanism: downregulation of the FA-BRCA pathway and conditional BRCAness

In normal cellular stress responses, DNA damage activates repair mechanisms. However, research by Mumblat et al. revealed a paradoxical phenomenon: in MPM cells treated with TTFields (specifically MSTO-211H and H2052 cell lines), despite a significant increase in DNA damage, key repair proteins were not activated but instead showed downregulated expression (9). Specifically, TTFields significantly reduced the expression levels of core components of the FA-BRCA pathway, including FANCA, FANCD2, FANCJ, and the key homologous recombination repair protein BRCA1.

This inhibition of the repair pathway delays the repair of DNA double-strand breaks. Studies by Giladi et al. in glioma models also confirmed that cells treated with TTFields exhibited significantly slower clearance of DNA fragments after irradiation compared to controls, and the recruitment of Rad51 repair proteins to damage sites was blocked (19). In MPM, this functional impairment of the FA-BRCA pathway induced by TTFields is termed “conditional BRCAness”. It is critical to distinguish this phenomenon from classical BRCAness. While classical BRCAness typically arises from intrinsic, irreversible genetic mutations (e.g., BRCA1/2 mutations) or stable epigenetic silencing within the tumor cells, conditional BRCAness represents a transient, non-genetic vulnerability. It is a dynamic state externally imposed by biophysical stress, characterized by the reversible transcriptional downregulation of homologous recombination repair components rather than structural genomic alterations. This externally modulated state offers a distinct therapeutic window, allowing for the application of synthetic lethality strategies in otherwise homologous recombination-proficient (wild-type) tumors. Unlike hereditary BRCA mutations, this state is imposed by an external physical field, is reversible and time-dependent, offering the possibility of implementing “synthetic lethality” strategies in wild-type BRCA tumors. Spatial transcriptomics data further support this view, showing extensive changes in gene expression profiles of cell cycle checkpoints and DNA repair in tumor tissues treated with TTFields, suggesting systemic regulation across multiple pathways (10) (**Table 1**).

**Table 1:** TTFields-induced alterations in subcellular structures and molecular pathways in malignant pleural mesothelioma.

Target structure/pathway	Key molecular players	Mechanism of action	Biological consequence	Reference
Mitotic Spindle	α/β-Tubulin dimers	Disruption of dipole alignment; interference with microtubule polymerization dynamics.	Mitotic arrest; chromosome segregation errors; mitotic catastrophe.	(7, 8)
Cytokinetic Ring	Septin 2, 6, 7 complexes	Perturbation of septin localization at the cleavage furrow; ectopic membrane blebbing.	Failure of cytokinesis; formation of multinucleated cells; aberrant mitotic exit.	(14, 15)
DNA Repair Machinery	FANCA, FANCD2, BRCA1 (Fanconi Anemia pathway)	Downregulation of protein expression; impaired recruitment of repair factors to DNA damage sites.	Impaired repair of DNA double-strand breaks (DSBs); induction of a state of “conditional BRCAness”; increased replication stress.	(9, 19)
Autophagy Regulation	AMPK, LC3-II	Induction of ER stress leading to AMPK-dependent autophagic flux.	Activation of a cytoprotective survival mechanism (potential therapeutic resistance).	(23)

This concept of induced molecular vulnerability is supported by independent investigations across diverse histological models. For example, Karanam et al. (20) demonstrated that TTFields elicit a state of conditional vulnerability in non-small cell lung cancer by downregulating BRCA1 signaling and compromising DNA double-strand break repair capacity, thereby sensitizing cells to ionizing radiation (20). More recently, comprehensive proteomic and phosphoproteomic analyses by Gao et al. (21) in glioblastoma models provided systemic evidence that TTFields broadly disrupt DNA repair and replication networks (21). Interestingly, this biophysical stress was shown to induce a compensatory upregulation of PARP1 and BRD4, suggesting a rational basis for concomitant targeted therapies utilizing PARP or BRD4 inhibitors.

### 3.3 Analysis of synergistic effects: differential sensitization of cisplatin vs. pemetrexed

The elucidation of the “conditional BRCAness” mechanism rationally explains the differential effects when TTFields are combined with different chemotherapeutic agents. In the clinical treatment of MPM, cisplatin or carboplatin combined with pemetrexed is the standard first-line regimen (2, 22).

#### 3.3.1 TTFields + platinum agents (synergy)

Platinum agents exert cytotoxicity by forming DNA interstrand cross-links (ICLs), and the FA-BRCA pathway is the key pathway for repairing ICLs. When TTFields downregulate this pathway, cancer cells cannot repair the DNA damage caused by platinum agents, leading to a lethal accumulation of damage. *In vitro* experiments confirmed that TTFields combined with cisplatin exhibit a significant synergistic effect (Synergy), where the combined efficacy is superior to the sum of the individual efficacies (9).

#### 3.3.2 TTFields + pemetrexed (additivity)

In contrast, pemetrexed primarily acts as an antimetabolite inhibiting nucleotide synthesis, a mechanism that does not directly rely on the repair function of the FA-BRCA pathway. Therefore, when TTFields are combined with pemetrexed, the effect is primarily additive (Additivity) rather than synergistic (9). This finding has important clinical implications: the combination of TTFields with platinum agents is not merely a physical superposition but a deep coupling driven by specific molecular mechanisms. This provides a solid molecular biological explanation for the excellent survival benefits achieved by TTFields combined with platinum-based chemotherapy in the STELLAR trial (6).

While much of the foundational mechanistic evidence regarding TTFields-induced “conditional BRCAness” has been derived from non-epithelioid models, its applicability to the predominant epithelioid subtype (~70% of cases) warrants specific attention. Emerging data from epithelioid-like models, such as the H2052 cell line, suggest that TTFields-mediated downregulation of FA-BRCA components is not confined to the more aggressive sarcomatoid or biphasic phenotypes. As detailed in **Table 2**, although the biological and histological gradients defined by BAP1 status and mesenchymal features may influence the net therapeutic impact, the induction of molecular vulnerability appears to be a conserved response to biophysical stress across the major MPM subtypes. Future investigations utilizing patient-derived epithelioid organoids will be essential to further substantiate this mechanism for the majority of patients.

**Table 2:** Histological heterogeneity and treatment response in malignant pleural mesothelioma.

Feature	Epithelioid subtype (~70%)	Non-epithelioid (sarcomatoid/biphasic) (~30%)
Genomic Characteristics	Lower mutational burden; frequent BAP1 loss	Higher mesenchymal markers; TP53 and NF2 alterations
Microenvironment	Often “immune-cold”; lower T-cell infiltration	Often “immune-hotter” or mesenchymal-rich
Chemotherapy Response	Higher objective response rates (ORR)	Often exhibits primary or rapid resistance
TTFields Mechanism	Emerging evidence of FA-BRCA downregulation (e.g., H2052)	Robust evidence of BRCAness and TNT disruption (e.g., MSTO-211H)
Combination Rationale	Potential “primer” to sensitize tumors to ICIs and Platinum	Strong potential for deep synergy with DNA-damaging agents

### 3.4 Clinical significance and limitations

Based on the above mechanisms, TTFields have been recommended by Chinese and international expert consensuses for the comprehensive treatment of unresectable MPM (26). The induction of a transient “conditional BRCAness” state provides a mechanism-based rationale for prioritizing platinum-based chemotherapy as the primary combinatorial partner with TTFields in MPM. Induced BRCAness not only enhances the efficacy of existing platinum-based chemotherapy but also suggests potential synergies between TTFields and other drugs targeting DNA repair defects (such as PARP inhibitors). However, current mechanistic studies focus primarily on specific cell line models; further exploration is needed regarding the heterogeneous distribution of this repair inhibition effect in patients and the existence of secondary resistance mechanisms.

## 4 The ecological remodeling — turning “cold” to “hot”

Beyond directly killing cancer cells through physical structural disruption and molecular pathway downregulation, another significant therapeutic potential of TTFields in MPM lies in their profound capacity to remodel the TME. This remodeling effect is not a unidimensional change. Still, it involves the “softening” of the extracellular matrix, the release of immunogenic signals, and the reconstruction of chemokine networks, collectively driving the phenotypic conversion of MPM from an immune “cold” tumor to a “hot” tumor. This ecological remodeling process provides a solid biological foundation for the combined application of TTFields with ICIs.

### 4.1 Remodeling and softening of the stromal barrier

**Figure 5:**
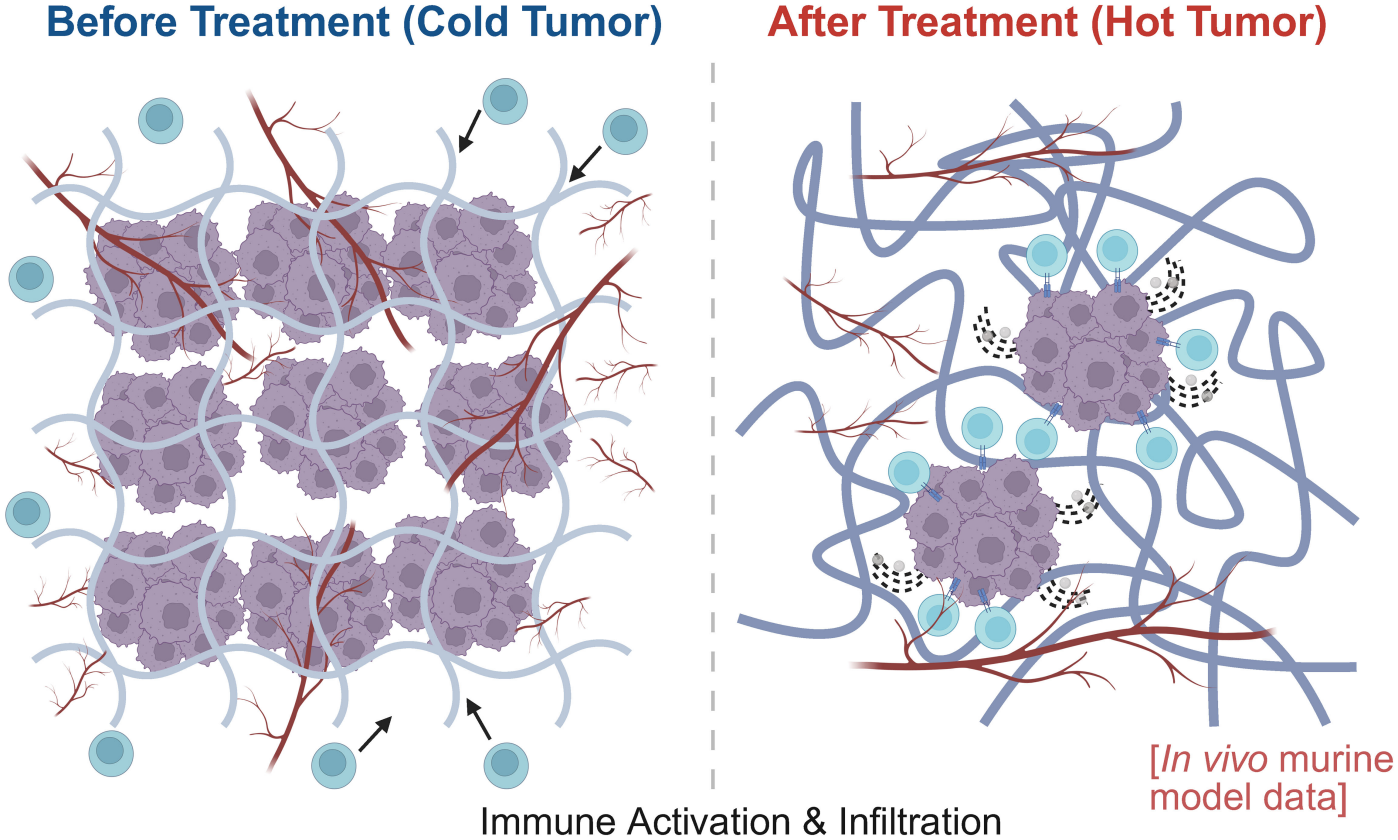
From “cold” to “hot”: the cascade of matrix softening and immune activation. This figure illustrates how TTFields reverse the immunosuppressive microenvironment of MPM. (1) Matrix Softening: The dense Tenascin C (TNC) network and high-density vessels (VEGF-driven) are reduced under electric fields, weakening the physical barrier. (2) Chemokine Storm: Tumor cells release chemokines CCL2, CCL8, CXCL9, and CXCL10. (3) Immune Infiltration: CD8+ T cells and Th1 cells follow the chemokine concentration gradient, penetrate the loosened matrix, and massively infiltrate the tumor core, converting the “cold” tumor into an immune “hot” tumor. (The depicted stromal softening and immune cell infiltration pathways are corroborated by recent in vivo murine model observations).

MPM is characterized by its dense desmoplastic stroma, a physical barrier that not only hinders the penetration of chemotherapeutic agents but also restricts immune cell infiltration (5). Spatial transcriptomic analysis revealed, for the first time, that TTFields treatment can significantly alter the stromal gene expression profile in MPM tumor tissues. Sarkari et al. observed in a murine MPM model that the expression levels of Tenascin C (TNC) and Vascular Endothelial Growth Factor A (VEGFA) were significantly downregulated in tumor regions after TTFields treatment (10). TNC is a multifunctional extracellular matrix glycoprotein that promotes tumor cell invasion and metastasis and is associated with a poor prognosis; VEGFA is a key factor driving angiogenesis and the formation of an immunosuppressive microenvironment (27) (**Figure 5**).

These molecular changes imply that TTFields physically “soften” the tumor stroma, weakening its function as a physical barrier. Combined with the previously mentioned TNT’s blockade effect, TTFields effectively disrupt the tumor’s defensive structures at both microscopic (intercellular connections) and macroscopic (stromal scaffold) levels, thereby opening physical channels for the deep infiltration of immune effector cells.

### 4.2 Induction of immunogenic cell death

Cell death induced by TTFields is not merely “silent” apoptosis but “ICD” capable of activating adaptive immune responses. Research by Voloshin et al. confirmed that TTFields treatment causes endoplasmic reticulum stress in cancer cells, promoting the translocation of calreticulin (CRT) from the endoplasmic reticulum to the cell surface, serving as an “eat-me” signal recognized by dendritic cells (DCs) (24). Simultaneously, damaged cells release High Mobility Group Box 1 protein (HMGB1) and adenosine triphosphate (ATP) into the extracellular space, acting as Damage-Associated Molecular Patterns (DAMPs) to activate the immune system.

**Figure 6:**
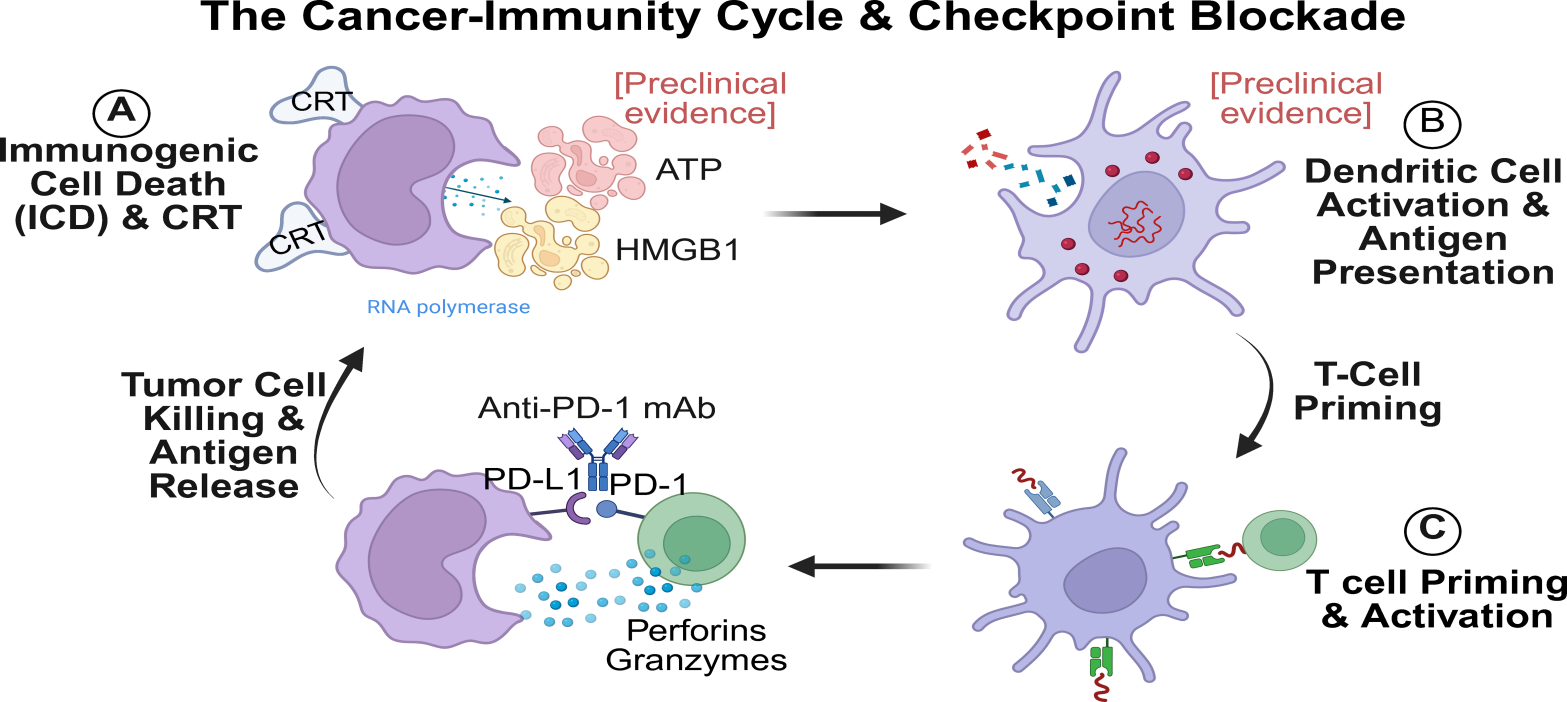
Systemic anti-tumor immune cycle driven by immunogenic cell death. **(A)** Signal Release: TTFields induce endoplasmic reticulum stress in cancer cells, leading to surface exposure of Calreticulin (CRT) and release of HMGB1 and ATP. **(B)** Dendritic Cell Activation: Dendritic Cells (DCs) recognize the CRT “eat-me” signal, phagocytose tumor antigens, and mature. **(C)** T-cell Activation & Synergy: Mature DCs activate T cells. In the TME, T cells recruited by TTFields may be inhibited by the PD-1/PD-L1 pathway; combining with anti-PD-1 antibodies releases this brake, achieving potent synergy between physical and immunotherapies. (The ICD induction and subsequent immune cycle activation steps are based on current preclinical evidence).

In *in vitro* co-culture experiments, cancer cells treated with TTFields significantly promoted DC maturation and phagocytosis, thereby enhancing the efficiency of antigen presentation (24). This process not only kills tumor cells *in situ* but, more importantly, initiates the systemic anti-tumor immune cycle, laying the foundation for subsequent T-cell activation (**Figure 6**).

### 4.3 Chemokine storm and T-cell recruitment

Inducing ICD alone is insufficient to guarantee T-cell homing to the tumor core; specific chemokine signals are required for guidance. Recent research by Lin et al. discovered that TTFields treatment can trigger a “chemokine storm” within the TME. Specifically, TTFields significantly upregulated the expression of key chemokines, including CCL2, CCL8, CXCL9, and CXCL10 (25). 1) CCL2/8: Primarily responsible for recruiting monocytes and macrophages. Although their role in tumors is dual-faceted, in specific contexts, they can promote the infiltration of antigen-presenting cells. 2) CXCL9/10: These are a more critical group of chemokines. By binding to the CXCR3 receptor on the surface of T cells, they specifically recruit CD8+ cytotoxic T lymphocytes (CTLs) and Th1 cells into the TME (25).

Experimental data showed that in mouse models treated with TTFields in combination with anti-PD-1 antibodies, the infiltration density of CD4+ and CD8+ T cells within the tumor tissue increased significantly, and the T cell exhaustion state was ameliorated (25). This suggests a dual mechanism where TTFields facilitate effective immune cell recruitment by simultaneously attenuating physical stromal barriers and upregulating chemotactic gradients.

Recent mechanistic investigations provide a definitive molecular link explaining this immune recruitment. As highlighted by Lee et al. (28), TTFields-induced nuclear membrane disruption leads to the accumulation of cytosolic micronuclei clusters (28). These fragments act as danger signals that dually activate the cGAS/STING pathway and the AIM2 inflammasome. This intracellular sensing cascade subsequently triggers the production of type 1 interferons and proinflammatory cytokines. The release of these signaling molecules into the tumor microenvironment plays a pivotal role in recruiting and activating natural killer cells, dendritic cells, and cytotoxic CD8+ T cells, thereby providing a clear molecular rationale for TTFields-mediated enhancement of antitumor immunity.

However, the dense, collagen-rich stroma characteristic of MPM acts as a formidable physical “fortress”, often rendering chemical gradients alone insufficient for profound immune infiltration. The biophysical remodeling induced by TTFields offers a potential solution to this barrier problem. As discussed previously, the downregulation of stromal components such as TNC suggests a structural loosening of the extracellular matrix. We hypothesize that this matrix softening operates synergistically with the induced chemokine storm. By physically altering the desmoplastic barrier to potentially reduce interstitial fluid pressure and tissue density, TTFields may facilitate the mechanical extravasation and subsequent penetration of recruited T-cells into the tumor core. This potential dual-action mechanism-simultaneously clearing the physical path and providing the chemical signal-presents a compelling theoretical rationale for the observed enhancement in intra-tumoral T-cell density (**Table 3**).

**Table 3:** TTFields-mediated remodeling of the tumor microenvironment and intercellular communication.

Microenvironmental component	Specific alterations observed	Functional impact on tumor biology	Immunological/clinical implication	Reference
Intercellular Communication	Suppression of Tunneling Nanotubes formation (specifically in biphasic MPM).	Blockade of intercellular mitochondrial rescue and oncogenic signal transfer (e.g., KRAS).	Physical isolation of cancer cells; prevention of the spread of adaptive resistance.	(10, 17)
Extracellular Matrix (ECM)	Downregulation of Tenascin C (TNC) and VEGFA expression.	Softening of the physical barrier; inhibition of angiogenesis and invasiveness.	Enhanced permeability for immune cell infiltration; reduction of immunosuppressive vascular signals.	(5, 10)
Immunogenic Signals	Surface exposure of Calreticulin; extracellular release of HMGB1 and ATP.	Induction of Immunogenic Cell Death (ICD); promotion of Dendritic Cell (DC) maturation and phagocytosis.	Priming of adaptive anti-tumor immunity; turning the tumor “visible” to the immune system.	(24)
Chemokine Network	Upregulation of CCL2, CCL8, CXCL9, and CXCL10.	Enhanced recruitment of cytotoxic T lymphocytes (CD8+) and Th1 cells into the tumor bed.	Sensitization to immune checkpoint inhibitors by reversing the “cold” tumor phenotype.	(25)

### 4.4 Clinical synergy: theoretical validation of physio-immunological combination therapy

The mechanisms described above—elimination of physical barriers, induction of ICD, and release of chemokines—collectively outline a potential logical model by which TTFields might assist in converting “cold” tumors into “hot” tumors. This ecological remodeling effect aligns with the mechanism of action of ICIs: ICIs primarily release T cell suppression. At the same time, TTFields increase the number of T cells within the tumor and improve their survival environment. The potential systemic antitumor immune response, often referred to as the abscopal effect when induced by localized physical therapies, is frequently restrained by the PD-1/PD-L1 axis; thus, combined PD-1 blockade may augment this systemic efficacy (24, 25, 29). For MPM patients, especially those with non-epithelioid subtypes that respond poorly to immunotherapy alone, this “physio-immunological coupling” strategy may be the key to overcoming resistance bottlenecks. CheckMate 743 established dual immunotherapy as a first-line status (4), and the addition of TTFields is expected to further improve the response rate and durability of this therapy, as observed in the STELLAR study, where the combination of physical therapy and systemic therapy can bring additive or even synergistic survival benefits (6) (**Table 4**).

**Table 4:** Rationale for combinatorial strategies based on TTFields-induced biological events.

Combinatorial partner	Target mechanism	Mechanistic rationale for combination	Interaction type	Clinical/preclinical evidence
Platinum Agents (Cisplatin/Carboplatin)	DNA cross-linking (ICL) formation.	TTFields-induced downregulation of the FA-BRCA pathway prevents the repair of platinum-induced DNA cross-links.	Synergy	(6, 9)
Antimetabolites (Pemetrexed)	Nucleotide synthesis inhibition.	Independent cytotoxic mechanisms leading to cumulative cell death without overlapping toxicities.	Additivity	(9)
Immune Checkpoint Inhibitors (Anti-PD-1/L1)	Reversal of T-cell exhaustion.	TTFields create a pro-inflammatory microenvironment (ICD + chemokines) that recruits T cells, which are then inhibited by ICIs.	Synergy	(24, 25)
PARP Inhibitors (Future Direction)	Synthetic lethality via PARP trapping.	Exploitation of TTFields-induced “conditional BRCAness” to trigger synthetic lethality in wild-type BRCA tumors.	Synergy (Predicted)	(9)

However, translating these synergistic approaches to complex clinical scenarios warrants careful consideration. For instance, in organ transplant recipients, PD-1 inhibition presents a “double-edged sword” by increasing the risk of allograft rejection, suggesting that concurrent immune modulation, such as mTOR pathway inhibition, might be required (30). Furthermore, emerging multi-omics insights indicate that systemic metabolic factors, particularly cholesterol homeostasis and SREBP-regulated lipid metabolism, play an integral role in shaping CD8+ T-cell effector functions and overall response to immunotherapy (31). The observation that TTFields can promote immunogenic cell death and orchestrate T-cell recruitment offers a translational basis for integrating this physical modality with ICIs. For clinical trial design, this suggests that patients presenting with immunologically “cold” characteristics might be rational candidates for exploring dual TTFields and ICIs regimens to overcome baseline resistance.

## 5 Clinical guidelines

As the biological mechanisms of TTFields in MPM—from the physical disintegration of subcellular structures to the remodeling of the immune microenvironment—are gradually elucidated, their clinical application value has been recognized by authoritative guidelines. However, to further break through efficacy bottlenecks, it is necessary to explore more precise combination therapy strategies and personalized diagnosis and treatment plans based on these newly discovered mechanistic atlases.

### 5.1 Clinical consensus and standardized application

Based on the positive results of the STELLAR phase II clinical trial, TTFields combined with pemetrexed and platinum-based chemotherapy showed significant survival benefits (median OS of 18.2 months) without increasing systemic toxicity (6). This evidence-based medicine has promoted the rapid inclusion of TTFields into international and Chinese clinical practice guidelines. The “Guidelines for Clinical Diagnosis and Treatment of Malignant Pleural Mesothelioma in China” released in 2023 and the “Consensus on Diagnosis and Treatment of Malignant Pleural Mesothelioma (2022, Hangzhou)” both explicitly recommend: for patients with unresectable MPM, in institutions with the conditions to carry out, first-line treatment may consider using TTFields combined with chemotherapy (pemetrexed + platinum) (Class II evidence, strong recommendation) (26, 32). The consensus points out that TTFields, as a convenient, low-side-effect novel comprehensive treatment modality, provides an essential option for MPM patients in addition to traditional chemotherapy and immunotherapy (32).

### 5.2 Safety profile and precision dosimetry

Safety is a significant advantage of TTFields as a long-term maintenance therapy. A meta-analysis of the safety of TTFields applied to the torso (involving NSCLC, MPM, etc.) showed that adverse events were mainly limited to skin reactions under the electrode patches (such as dermatitis), mostly mild to moderate (Grade 1-2), with no significant increase in systemic toxicity (such as myelosuppression or cardiotoxicity) observed (33). This good tolerability provides a safety window for the simultaneous use of TTFields with dual immunotherapy or multi-drug chemotherapy, avoiding treatment interruptions due to overlapping toxicities.

In dosimetry, precise setting of physical parameters appears to be a critical factor for optimizing efficacy. *In vitro* studies confirmed that the sensitivity of MPM cells to TTFields is frequency-specific, with 150 kHz identified as the optimal inhibitory frequency, distinct from that of glioblastoma (200 kHz) (34). Furthermore, computational models constructed using the Finite Element Method (FEM) indicate that the spatial distribution of the electric field is significantly influenced by thoracic anatomical structures and tissue dielectric properties (35). Therefore, future clinical practice may need to rely more on personalized TPS to ensure optimal coverage of the tumor target volume with field strength.

### 5.3 Positioning TTFields in the immunotherapy era

With the pivotal CheckMate 743 trial establishing nivolumab plus ipilimumab as a standard first-line treatment for unresectable MPM, the clinical landscape has fundamentally shifted toward an immunotherapy-dominated era (4). Within this contemporary context, the clinical positioning of TTFields requires re-evaluation.

Currently, while direct clinical data on the combination of TTFields and immune checkpoint inhibitors (ICIs) in MPM remains nascent, the physio-biological remodeling mechanisms discussed previously offer a strong theoretical rationale for this combination. The capacity of TTFields to induce immunogenic cell death (ICD) and upregulate specific chemokines (e.g., CXCL9/10) theoretically addresses a major limitation of ICIs in MPM: the “immune-cold” microenvironment. By physically perturbing the tumor architecture and subsequently recruiting T-cells into the tumor bed, TTFields may act as a primer, rendering previously unresponsive tumors sensitive to PD-1/PD-L1 blockade.

From a clinical perspective, this combination strategy may hold particular relevance for specific patient populations. For instance, while sarcomatoid and biphasic subtypes demonstrate notable benefits from immunotherapy, patients with the epithelioid subtype often exhibit more constrained responses to ICIs alone. Future research should investigate whether the immunomodulatory effects of TTFields can preferentially enhance ICI efficacy in these specific histological subgroups.

Moving forward, designing prospective clinical trials that combine TTFields with current standard-of-care immunotherapies is a priority. These studies will be essential to transition the promising preclinical synergistic effects into validated, evidence-based clinical guidelines.

## 6 Future perspectives — beyond the status quo

**Figure 7:**
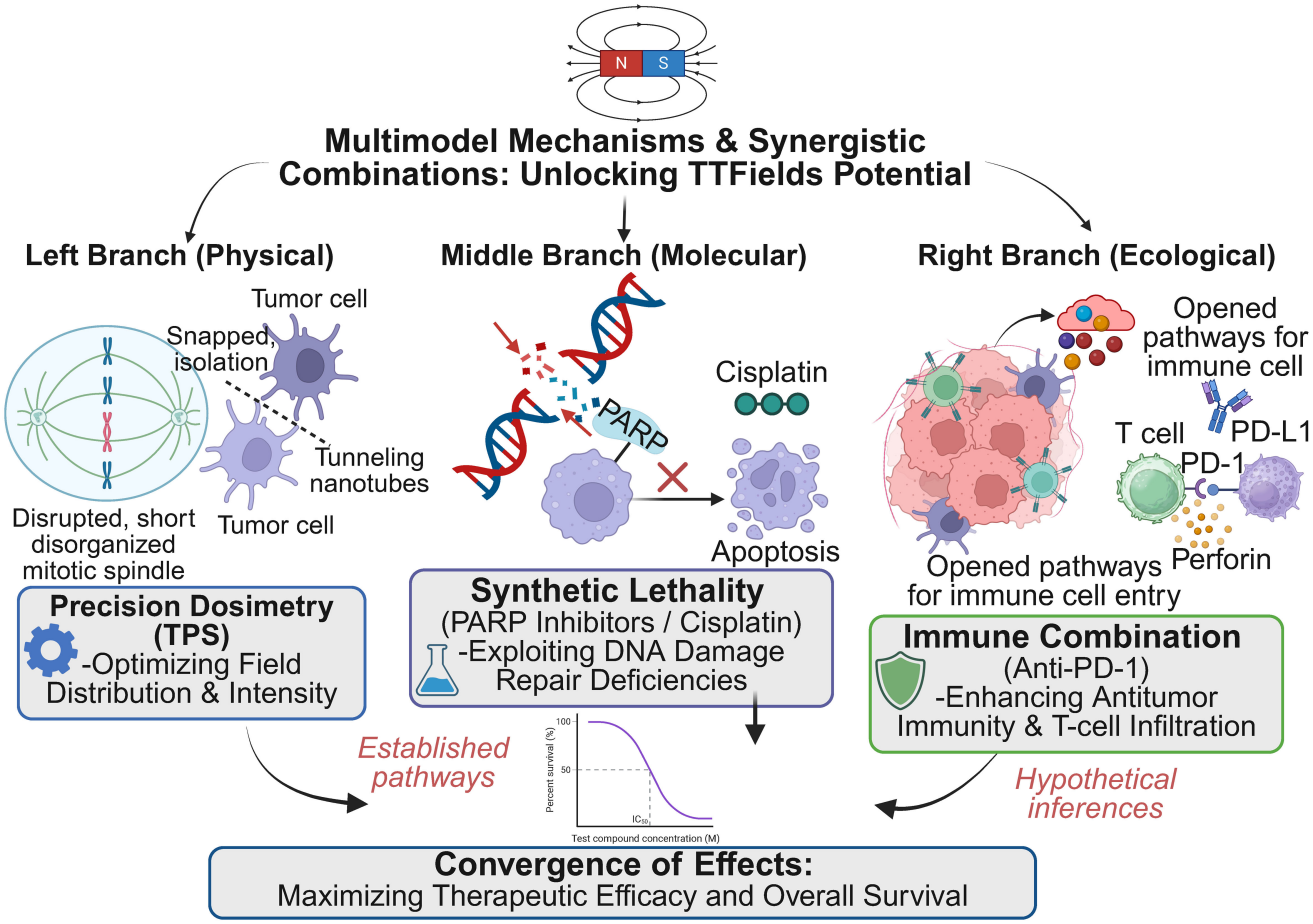
Atlas of multidimensional biophysical remodeling and future combination therapy roadmap. This panoramic diagram summarizes the three dimensions of TTFields action in MPM and corresponding future clinical translation strategies. (1) Physical Dimension: Disruption of mitosis and TNTs → Strategy: Precision treatment planning and physical parameter optimization. (2) Molecular Dimension: Downregulation of FA-BRCA pathway and induced BRCAness → Strategy: Combination with PARP inhibitors (Synthetic Lethality 2.0) and platinum agents. (3) Ecological Dimension: Immunogenic cell death and microenvironment remodeling → Strategy: Combination with immune checkpoint inhibitors and autophagy inhibitors. (Left Branch and Middle Branch represent established molecular and physical interactions based on existing literature. Right Branch illustrate hypothesized therapeutic synergies and potential clinical translation routes).

Although TTFields have established an essential role in the comprehensive treatment of MPM, their potential biological effects and clinical application boundaries remain underexplored. Based on the “multidimensional biophysical remodeling” atlas constructed in this paper, future research is suggested to focus on further leveraging the molecular vulnerabilities and microenvironmental changes induced by TTFields, and to explore the possibility of breakthroughs in efficacy through precise combination strategies and biomarker development (**Figure 7**).

### 6.1 Synthetic lethality 2.0: targeting induced DNA repair defects

Given that TTFields have been confirmed to induce a state of “conditional BRCAness” in wild-type MPM cells by downregulating the FA-BRCA pathway (9), future clinical translational research is suggested to focus on exploring the therapeutic potential of this molecular vulnerability. Particularly, poly (ADP-ribose) polymerase (PARP) inhibitors have demonstrated significant efficacy in BRCA-mutated tumors but often have limited effects in wild-type tumors. Theoretically, the DNA repair-deficient environment created by TTFields may render wild-type MPM sensitive to PARP inhibitors, as in BRCA-mutated tumors (9). Therefore, initiating preclinical and early clinical trials of TTFields combined with PARP inhibitors (such as olaparib and niraparib) is a critical step toward validating the “Synthetic Lethality 2.0” strategy. Furthermore, exploring the combination of TTFields with other DDR-targeting drugs (such as ATR or WEE1 inhibitors) may further expand the therapeutic window.

### 6.2 Metabolic intervention: blocking protective autophagy

When cells are subjected to the physical strike of TTFields, they do not die passively but initiate AMPK-dependent autophagy as an adaptive survival mechanism (23). This protective autophagy may attenuate the cytotoxic efficacy of TTFields to some extent, becoming a potential source of resistance. Future research should focus on validating the synergistic effect of autophagy inhibitors (such as chloroquine and its derivatives, or novel, specific autophagy inhibitors) combined with TTFields (23). By simultaneously delivering a physical strike (TTFields) and blocking the metabolic escape route (autophagy inhibition), it is expected to overcome adaptive resistance and achieve deeper tumor remission.

### 6.3 Development of spatial biomarkers and precision stratification

To transition the proposed biophysical remodeling atlas from a theoretical model to a clinically actionable tool, future clinical trials should prioritize the integration of specific, measurable biomarker panels. Validating the sequential efficacy of this cascade requires tracking indicators across multiple biological levels. First, early responses to biophysical stress could be monitored systemically via markers of immunogenic cell death, such as the release of circulating HMGB1 or the surface exposure of Calreticulin on tumor cells. Second, to substantiate the hypothesized “chemokine storm”, quantifying specific local and systemic chemokines-most notably CXCL9 and CXCL10-will be critical to determine whether the tumor is actively generating recruitment signals. Finally, at the terminal ecological level, comprehensive spatial profiling of the tumor microenvironment is necessary. This should involve evaluating not only the absolute counts and infiltration density of CD8+ T-cells but also assessing their functional state (e.g., mapping PD-1 or TIM-3 expression profiles). Systematically tracking these specific metrics will provide the essential quantitative evidence needed to evaluate whether the application of TTFields is effectively translating into a functional anti-tumor immune response in individual patients.

### 6.4 Dynamic optimization and personalization of physical parameters

Although the standard treatment frequency for MPM is currently set at 150 kHz (34), the tumor’s physical properties (e.g., cell size, dielectric constant) may evolve during treatment. Future research can explore whether dynamic adjustment of electric field frequency or intensity based on tumor response to treatment is needed. Furthermore, personalized TPS incorporating high-resolution imaging and finite element modeling will enable more precise targeting of the maximum field strength to highly active regions of heterogeneous tumors, thereby improving treatment efficiency and reducing potential impacts on surrounding normal tissues (35).

## 7 Limitations

Although this paper constructs a “multidimensional biophysical remodeling” atlas that provides a new perspective on the mechanism of action of TTFields in MPM, the existing chain of evidence still has significant limitations in translating basic research into clinical application. These deficiencies not only reflect gaps in current research but also guide the direction of future validation studies.

### 7.1 Translational gaps in preclinical models

Current evidence regarding the physical blockade of intercellular communication by TTFields relies predominantly on 2D *in vitro* cell culture models. While significant inhibition of TNT formation by TTFields has been observed in biphasic MPM cell lines, generalizing the “physical isolation” theory to the human TME requires profound caution. MPM is pathologically characterized by a highly dense, desmoplastic, and scarred stromal tissue. In a true *in vivo* pleural environment, the significant mechanical tension and spatial hindrance exerted by this collagen-rich extracellular matrix present a formidable physical barrier. This dense stroma might naturally restrict or even prevent the extensive formation and maintenance of fragile actin-based nanotubes compared to unconstrained laboratory dishes. Consequently, the actual prevalence of these networks in patients, and the subsequent clinical impact of severing them with TTFields, remains a critical translational gap that future 3D matrix models must address (10). Furthermore, research on the molecular mechanism of “induced BRCAness” has mainly focused on specific non-epithelioid cell lines (such as MSTO-211H and H2052) (9). Considering the high histological and molecular heterogeneity of MPM (5), whether this downregulation of DNA repair pathways is universally present in all subtypes (especially epithelioid subtypes) requires further validation using more patient-derived primary cells or organoid models.

### 7.2 Limitations in clinical trial design

The core clinical evidence supporting the application of TTFields in MPM comes from the STELLAR trial. However, this was a single-arm, non-randomized phase II clinical study, and its efficacy was evaluated against historical control data (6). Although the study showed encouraging survival benefits, the lack of a randomized controlled trial (RCT) design inevitably introduces the possibility of selection bias, and it is difficult to completely rule out the influence of “patient effects” on survival. Moreover, with the CheckMate 743 study establishing dual immunotherapy (nivolumab combined with ipilimumab) as the new standard for first-line treatment of MPM (4), current clinical data for TTFields are mainly based on combination with chemotherapy. The lack of head-to-head comparisons or high-level clinical data on the combined use of TTFields with the current standard immunotherapy regimen limits the clarity of its positioning in the new era dominated by immunotherapy.

### 7.3 Biomarker and dosimetric challenges

Regarding biomarkers, although preclinical studies have proposed ICD markers such as HMGB1 release and calreticulin exposure (24), as well as spatial transcriptomic features as potential predictors (10), these indicators have not yet been validated on a large scale in routine clinical testing. There is currently a lack of validated predictive biomarkers to screen beneficiary populations precisely. In terms of dosimetry, the complexity of thoracic anatomical structures (such as lung air, pleural effusion, and conductive differences) significantly impacts the electric field distribution. Simulation studies indicate that the distribution of electric field intensity within tissues is highly non-uniform (35). In clinical practice, monitoring in real time and ensuring that the tumor target volume is always covered by therapeutic field strength remains an unresolved technical challenge, which may lead to variations in efficacy across patients.

### 7.4 Publication bias and limits of extrapolation

When evaluating the current mechanistic landscape of TTFields, the potential for publication bias and selective reporting merits critical consideration. Preclinical studies inherently tend to report positive, synergistic mechanisms, while negative or neutral data regarding TTFields’ cellular interactions remain largely underrepresented in the literature. Furthermore, a substantial portion of the foundational biophysical understanding of TTFields is extrapolated from glioblastoma models, which operate at an optimal frequency of 200 kHz. Given the pronounced morphological and dielectric differences between glial cells and pleural mesothelial cells (which demonstrate optimal response at 150 kHz), directly translating these biophysical paradigms across distinct tumor microenvironments warrants cautious interpretation.

### 7.5 Contradictory findings and adaptive resistance

To maintain scientific objectivity, it is essential to acknowledge findings that diverge from the proposed synergistic cascade. While the current framework emphasizes processes like ICD and induced BRCAness, concurrent biophysical stress also activates robust cellular survival programs. For instance, evidence indicates that TTFields can trigger AMPK-dependent protective autophagy, a pro-survival mechanism that may counteract therapeutic efficacy and foster adaptive resistance (23). Additionally, while the physical alteration of the dense stroma is hypothesized to facilitate immune infiltration, such microenvironmental disruption theoretically risks releasing sequestered pro-tumorigenic cytokines under certain conditions. These competing biological responses suggest that the overall efficacy of TTFields *in vivo* is a dynamic net result of opposing forces, underscoring the complexity of its biological impact rather than a strictly unidirectional anti-tumor progression.

## 8 Conclusion

In summary, this review, based on existing evidence, proposes the potential multidimensional mechanisms of action of TTFields in MPM. Current preclinical studies and clinical data suggest that the effects of TTFields are not limited to simple mitotic arrest but involve comprehensive regulation of the tumor’s physical structure and biological microenvironment.

First, at the physical structural level, in addition to classical microtubule interference, TTFields demonstrate the potential to disrupt intercellular communication structures. *In vitro* studies have shown that TTFields can inhibit the formation of “TNTs” between MPM cells; the disruption of this physical connection may limit metabolic support and signal communication between cancer cells to some extent (10). Second, at the molecular level, downstream effects of physical stress lead to downregulation of the FA-BRCA pathway. This “conditional BRCAness” state increases the sensitivity of wild-type MPM cells to DNA-damaging agents (such as cisplatin), providing a molecular biological explanation for the combined application of TTFields and chemotherapy (9). Finally, at the microenvironmental level, the release of signals associated with ICD induced by TTFields treatment, along with the upregulation of chemokine expression, may improve the local microenvironment’s immune status and promote the infiltration of effector T cells (24).

Combinatorial Rationale: TTFields demonstrate biological plausibility for synergistic use with DNA-damaging agents (by exploiting induced DNA repair defects) and immunotherapies (by mitigating the immunosuppressive matrix). Application Precision: The therapeutic impact at the cellular level is highly dependent on continuous exposure. Thus, rigorous patient compliance and anatomically precise field distribution via TPS remain critical parameters for clinical success. Future Trajectories: Clinical research should increasingly focus on assessing the safety and efficacy of TTFields in combination with current dual-immunotherapy standards, moving beyond traditional chemotherapy-only backbones.

Collectively, the clinical benefits of TTFields demonstrated in MPM treatment may result from the combined action of the aforementioned physical blockade, molecular sensitization, and immune regulatory mechanisms. This mechanistic framework, integrating cytoskeletal interference with microenvironmental remodeling, provides a theoretical basis for exploring future strategies that combine TTFields with ICIs or DNA repair inhibitors.
